# Acknowledgement

**Published:** 2023

We are extremely grateful to the following members of the medical profession from Pakistan and Overseas for sparing their precious time to review the manuscript published in the Issue of September - October 2023.

**Table:** 

Reviewer’s Name	(Manuscript Reviewed)	Reviewer’s Name	(Manuscript Reviewed)	Reviewer’s Name	(Manuscript Reviewed)
Abdul Haque, ***Pakistan***		Madeeha Rehan, ***Pakistan***		Salhah Alsulami, ***Saudi Arabia***	
Adeela Shahid, ***Pakistan***		Mahnaz Raees, ***Pakistan***		Sameen Afzal Junejo, ***Pakistan***	2
Amjad Khan, ***South Korea***		Mahwish Arooj, ***Pakistan***		Sana Zeeshan, ***Pakistan***	
Anita Haroon, ***Pakistan***		Maseeh Uz Zaman, ***Pakistan***		Sehar Khaliq, ***Pakistan***	
An-jun Li, ***China***		May Khalil Ismail, ***UAE***		Seyda Kauser Ali, ***Pakistan***	
Anupama Bondili, ***UAE***		Md. Abdus Salam, ***Malaysia*** 1		Shahida Mushtaq, ***Pakistan***	
Asma Naseer Cheema, ***USA***		Mehwash Kashif, ***Pakistan***		Shan-shan Zhu, ***China***	
Ayse Rabia Senkaya, ***Turkey***		Memoona Mansoor, ***Pakistan***		Song Zhang, ***China***	
Azra Mahmud, ***Pakistan***		Meng Chen, ***China***		Summera Aslam, ***Pakistan***	
Bader Faiyaz Zuberi, ***Pakistan***	2	Ming-hui Hou, ***China***		Sundus Tariq, ***Pakistan***	
Bin Liu, ***China***		Mohammad Asim Mehboob, ***Pakistan***		Syed Fawad Mashhadi, ***Pakistan***	
Dong Xing, ***China***		Muhammad Ali Syed, ***Pakistan***		Syed Riazul Hasan, ***Pakistan***	
Ejaz Ahmed Vohra, ***Pakistan***		Muhammad Imran Naseer, ***Saudi Arabia***		Tang Wenyuan, ***China***	
Emrah Beyan, ***Turkey***		Muhammad Irfan, ***Pakistan***		Tayyaba Gul Malik, ***Pakistan***	
Faaiz Ali Shah, ***Pakistan***		Muhammad Mussaffa Butt, ***Pakistan***		Tayyiba Wasim, ***Pakistan***	
Faisal Nazeer Hussain, ***Pakistan***	2	Muhammad Shahid Nadeem, ***Saudi Arabia***		Tian-yu Han, ***China***	
Farnaz Zahoor, ***Pakistan***		Mukhtiar Baig, ***Saudi Arabia***		Ujala Sajid, ***Pakistan***	
Fazal I Wahid, ***Pakistan***		Murat Kutlu, ***Turkey***		Vicar Solomon, ***Pakistan***	
Gauthaman Kalamegam, ***India***		Musarrat Riaz, ***Pakistan***	6	Wajid Jawaid, ***Pakistan***	
Gohar Wajid, ***Egypt***		Muzaffer Sanci, ***Turkey***		Waris Qidwai, ***Pakistan***	
Goknur Yorulmaz, ***Turkey***		Nasir Khokar, ***Pakistan***		Xian-yi Liu, ***China***	
Gulnaz Shafqat, ***Pakistan***		Naveed Ali Shair, ***Pakistan***		Xiao-Li Li, ***China***	
Hai-bo-Li, ***China***		Na-ying Zuo, ***China***		Xiao-min Meng, ***China***	
Hai-tao Cao, ***China***		Nazeer Ahmed, ***Pakistan***		Xin Li, ***China***	
Hala Hisham Mosli, ***Saudi Arabia***		Nazish Imran, ***Pakistan***	2	Yalcup Tomack, ***Turkey***	
Hong-wei Zhang, ***China***		Nazish Saqlain, ***Pakistan***	2	Yang Liu, ***China***	
Hong-zhen Zhang, ***China***		Nisreen Amayiri, ***Jordan***		Yan-li Zhang, ***China***	
Hui-qun Jia, ***China***		Omema Saleem, ***Pakistan***		Ying Hu, ***China*** 2	
Ibrahim Qaddoumi, ***USA***		Ping Wang, ***China***		Ying-xin Gong, ***China***	
Ijaz Hussain, ***Pakistan***		Qing-huai Li, ***China***		Younis Abdelwahab Skaik, ***Germany***	
Irfan Ali, ***Pakistan***		Qinlu Hunag, ***China***		Youxiong Que, ***China***	
Jia-rong Wang, ***China***		Raja Irfan Qadir, ***Pakistan***		Yuan Yue, ***China***	
Jing-qin Liu, ***China***		Ramesh Kumar, ***Pakistan***		Zafar Ahmed, ***Pakistan***	
Jinlian Qin, ***China***		Rehana Rehman, ***Pakistan***		Zeeshan Khan, ***Pakistan***	
Li Li, ***China***		Sadaf Ahmed, ***Pakistan***		Zhang Guixin, ***China***	
Lijun Feng, ***China***		Saima Batool, ***Pakistan***		Zhi-hong Li, ***China***	
Ling Zhang, ***China***				Zubia Masood, ***Pakistan***	
Lin-lin Feng, ***China***					
Lin-xia Li, ***China***					

